# Cellular heterogeneity and immune responses to smut pathogen in sugarcane

**DOI:** 10.1111/pbi.70084

**Published:** 2025-04-09

**Authors:** Shoujian Zang, Qibin Wu, Dongjiao Wang, Zhenxiang Li, Tingting Sun, Xinlu Sun, Tianzhen Cui, Yachun Su, Haifeng Wang, Youxiong Que

**Affiliations:** ^1^ National Key Laboratory for Tropical Crop Breeding Institute of Tropical Bioscience and Biotechnology/Sanya Research Institute, Chinese Academy of Tropical Agricultural Sciences Sanya Hainan China; ^2^ Key Laboratory of Sugarcane Biology and Genetic Breeding, Ministry of Agriculture and Rural Affairs, National Engineering Research Center for Sugarcane College of Agriculture, Fujian Agriculture and Forestry University Fuzhou China; ^3^ State Key Laboratory for Conservation and Utilization of Subtropical Agro‐Bioresources, Guangxi Key Lab for Sugarcane Biology College of Agriculture, Guangxi University Nanning China

**Keywords:** sugarcane, *Sporisorium scitanmineum*, scRNA‐seq, cell differentiation, gene function

Sugarcane (*Saccharum* spp.) is a globally significant crop, valued for its contribution to the production of sugar and bioenergy. Despite its economic and industrial importance, sugarcane is highly vulnerable to smut disease caused by *Sporisorium scitamineum*, a major fungal pathogen that substantially reduces yield and quality (Wu *et al*., 2024). The complexity of the sugarcane genome, characterized by polyploidy, heterozygosity, and large genome size, has hindered the identification of resistance‐related genes and limited progress in genomic research (Wu *et al*., 2024). It is still unclear how genes in this species are expressed at the cellular level, especially in the case of smut pathogen infection. To address this knowledge gap, we optimized a sugarcane protoplast extraction method and successfully performed single‐cell RNA sequencing (scRNA‐seq), providing a high‐resolution scRNA‐seq atlas of gene expression during smut pathogen infection. This work uncovers key molecular mechanisms underlying the sugarcane–smut pathogen interaction, offering novel insights into plant immunity.

Our study focused on smut‐resistant (YT93‐159) and smut‐susceptible (ROC22) sugarcane cultivars. We observed that smut pathogen proliferation was significantly higher in ROC22 buds than in YT93‐159 after inoculation (Figure S1A,B). By refining the protoplast isolation process, we obtained high‐quality single cells from sugarcane buds (Figure S1C). Through scRNA‐seq analysis at 0 and 2 days post‐inoculation on both ROC22 and YT93‐159 buds (Figure 1a), we classified sugarcane bud cells into 17 distinct clusters (Table S1), subsequently grouped into 10 cell populations by using reported marker genes and plant scRNA‐seq databases (Figure 1b; Table S2). These populations included cortex (Co), meristem (Mr), epidermal (Ep), mesophyll (Ms), bundle sheath (Bu), stele (St), proliferating (Pr), vascular (Va), guard (Gu), and unknown (UK) cells (Figure 1c; Figure S2). Marker gene profiling confirmed the identity of these clusters, establishing a comprehensive cell atlas that served as a robust foundation for further functional studies (Figure S3). To facilitate further research, we constructed a cluster‐specific marker gene library, enabling precise categorization of sugarcane tissues into specific cell types (Table S3). For example, epidermal cells (clusters 2, 6, 12, and 13) were identified by high expression of *KCS*, *GER7*, *LTP*, and *ABCG11* (Satterlee *et al*., 2020), while guard cells (cluster 11) were marked by *FAMA* and *MYB60* (Guo *et al*., 2022). Meristem clusters (clusters 1 and 5) expressed *GA2OX6* and histone genes (*HIS2A* and *H2B*) (Cao *et al*., 2023), and vascular cells (clusters 10 and 16) showed markers like *XCP2* and *CCoAOMT1*, the latter linked to lignin production (Li *et al*., 2021). Cluster 7, characterized as bundle sheath cells, contained photosynthesis‐related genes such as *rbcL* and *psbB* (Satterlee *et al*., 2020; Stoppel *et al*., 2011). Proliferating cells (cluster 9) exhibited high levels of mitotic and cell cycle‐related genes such as *CYCB1‐1* and *CYCB2‐2*. Overall, these clusters were classified into 10 distinct cell types (Figure 1b). This single‐cell atlas, along with the marker gene library, establishes a valuable resource and a robust foundation for advancing functional characterization and genetic engineering of key genes in sugarcane (Figure S4).

**Figure 1:**
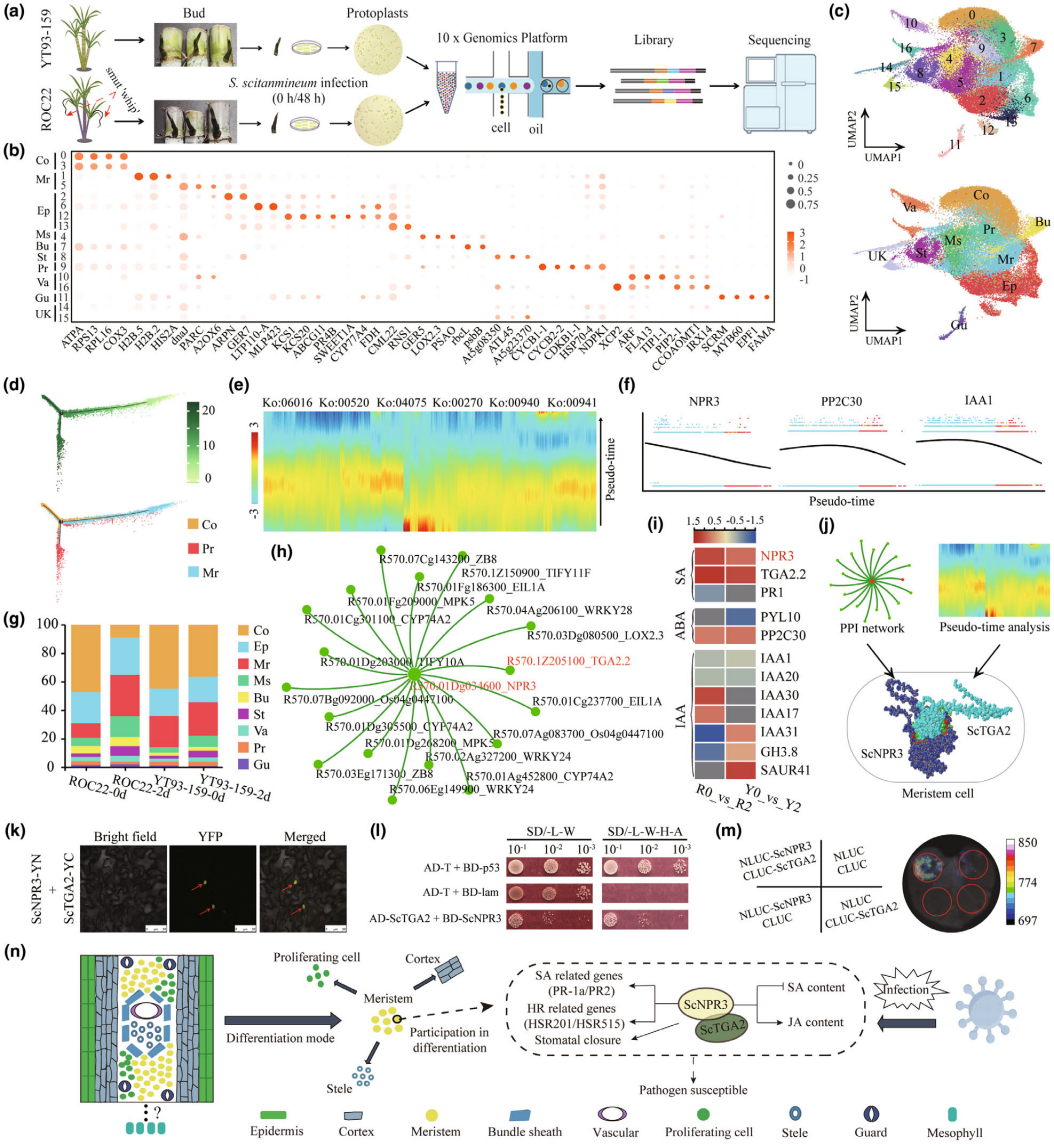
Single‐cell atlas and regulatory mechanisms in sugarcane. (a) Overview of the sugarcane scRNA‐seq workflow. (b) Expression patterns of representative cell‐specific marker genes identified 10 major cell types. (c) UMAP visualization of the 10 major cell clusters in pooled samples from ROC22 and YT93‐159. (d) Pseudo‐temporal trajectories of cortex, proliferating, and meristem cells. (e) The relative expression levels of pseudo‐time‐dependent genes along the trajectory of meristem cells and the KEGG enrichment pathways of significantly altered genes. (f) Representative genes related to plant hormone signal transduction were selected to illustrate their expression patterns before and after cell differentiation. (g) Relative proportion of each cell type in ROC22 and YT93‐159 at 0 d and 2 d post‐inoculation with smut pathogen. (h) Protein–protein interaction network of NPR3 with up‐regulated genes in the meristem cells in R0_vs_R2 and Y0_vs_Y2. (i) Heatmap showing the Log_2_ (fold change) differences of genes related to plant hormone signal transduction in R0_vs_R2 and Y0_vs_Y2 in the meristem cells. (j) The interaction between ScNPR3 and ScTGA2. (k) BiFC analysis of ScNPR3 and ScTGA2 interaction in *N. benthamiana* leaves. (l) ScNPR3 interacted with ScTGA2 in yeast. (m) LUC analysis of the interaction between ScNPR3 and ScTGA2 in *N. benthamiana* leaves. (n) A schematic diagram for cell structure in sugarcane buds and the regulatory model involving ScNPR3 in the meristem differentiation.

We identified the meristem as a critical site of differentiation and immune response. Pseudo‐temporal trajectories analysis revealed that meristem cells differentiated into cortex and proliferating cells (Figure 1d; Figure S5A). To better understand this differentiation process, we examined the expression patterns of genes with altered transcriptional regulation during the transition from meristem cells to cortex and proliferating cells along the pseudo‐time axis (Figure 1e; Table S4). These genes were significantly enriched in stress‐related pathways, including MAPK signalling, phytohormone signalling, flavonoid biosynthesis, and phenylpropanoid biosynthesis (Figure S5B). Particularly, several genes like *NPR3*, *PP2C30*, *IAA1, PR1*, *SnRK2*, and *SAUR* exhibited dynamic expression patterns along the pseudo‐time axis, suggesting their involvement in meristem differentiation and defence response (Figure 1f; Figure S5C,D). Cell‐to‐cell heterogeneity played a critical role in sugarcane's response to smut pathogen infection. Cell ratios of YT93‐159 and ROC22 differed significantly at 0 d and 2 days post smut pathogen infection (Figure 1g). Venn diagram analyses revealed distinct patterns of up‐regulated and down‐regulated genes across six major cell types, reflecting unique biological responses between the two cultivars (Figure S6). In ROC22, up‐regulated genes were enriched in oxidative phosphorylation and ribosome activity pathways, whereas YT93‐159 exhibited a focus on alpha‐linolenic acid metabolism, photosynthesis, and plant hormone signalling. Cell‐specific responses were evident, with mesophyll cells prioritizing photosynthesis and fatty acid metabolism (Figure S6C), and stele cells emphasizing protein processing and oxidative phosphorylation (Figure S6E). Notably, YT93‐159 exhibited a higher proportion (2.13 times higher) of meristem cells in non‐inoculated plants compared to ROC22 (Table S5). After inoculation, meristem cell numbers increased significantly in ROC22 but only moderately in YT93‐159 (Figure 1g). In the ROC22 meristem, up‐regulated genes primarily activated pathways related to oxidative phosphorylation and the citrate cycle, while in YT93‐159, they were enriched in alpha‐linolenic acid metabolism and plant hormone signalling pathways (Figure S7). Analysis of gene expression changes in plant hormone signalling indicated that genes such as *NPR3* and *PP2C30*, which decreased along the pseudo‐time axis (Figure 1f), were up‐regulated in both cultivars after inoculation (Figure 1i). This highlighted their potential involvement in stress response. Protein–protein interaction (PPI) network analysis further identified NPR3 as a central node interacting with proteins like TGA2.2 and WRKY transcription factors, mediating the trade‐off between growth and defence under stress conditions (Figure 1h).

We further investigated the function of *NPR3* by cloning its coding sequence from ROC22, named *ScNPR3*. It contained the NPR1‐like‐C domain and was phylogenetically related to other *NPR* genes (Figure S8A–C). *ScNPR3* was constitutively expressed in various tissues and induced by MeJA, ABA, and SA stress (Figure S8D). Overexpression of *ScNPR3* in *Nicotiana benthamiana* (Figure S8E–G) exhibited reduced pathogen resistance, with increased H_2_O_2_ and JA levels but decreased SA content after inoculation (Figure S9A–E). RNA‐seq analysis revealed that the DEGs in *ScNPR3*‐overexpressing plants were not enriched in stress‐related pathways, partially explaining their reduced defence (Figure S9F–H). Finally, we validated the interaction between ScNPR3 and ScTGA2 as predicted by the PPI network (Figure 1j). ScTGA2, a typical BZIP family transcription factor, contains both BZIP and DOG1 domains (Figure S10A). Subcellular localization analyses uncovered that ScNPR3 and ScTGA2 are localized in the nucleus (Figure S10B). To confirm their interaction, we explored Y2H, BiFC, and LUC assays, which consistently demonstrated that ScNPR3 interacted with ScTGA2 to form a protein complex (Figure 1k–m, Figure S10C). These findings suggest that ScNPR3 negatively regulates plant defence mechanisms by interacting with ScTGA2 (Figure 1n).

In summary, this study provides the first scRNA‐seq atlas in sugarcane and reveals key molecular events underlying the sugarcane‐smut pathogen interaction. The identified ScNPR3‐ScTGA2 regulatory mechanism provides a foundation for improving disease resistance in sugarcane and offers insights into plant stress responses.

## Conflict of interest

The authors have declared no conflict of interest.

## Author contributions

Y.Q., H.W., and Q.W. conceived and designed the project. S.Z., D.W., T.S., and Y.S. analysed the data. S.Z., T.C., Z.L., and X.S. performed the experiments. S.Z. and Q.W. wrote the manuscript draft. Y.Q., H.W., and Q.W. revised the manuscript.

## Supporting information


**Figure S1–S10** Supplementary Figures.

**Table S1–S6** Supplementary Tables

## Data Availability

The scRNA‐seq and RNA‐seq data have been deposited at Beijing Institute of Genomics Data Center (http://bigd.big.ac.cn). Accession numbers are PRJCA023019 and PRJCA027166.
