# Supplementary material for: Cellular heterogeneity and immune responses to smut pathogen in sugarcane
Source: Plant Biotechnol J. 2025 Apr 9;23(7):2608–10. doi: 10.1111/pbi.70084 (PMC12205859; doi:10.1111/pbi.70084)
Supplement: Supplementary file 1 — Figure S1–S10 Supplementary Figures. [file PBI-23-2608-s001.docx]

**Supplemental materials and methods**

**Plant materials and treatments**

Two sugarcane cultivars, YT93-159 (smut resistant) and ROC22 (smut susceptible) were provided by the Key Laboratory of Sugarcane Biology and Genetic Breeding, Ministry of Agriculture and Rural Affairs, Fujian Agriculture and Forestry University (FAFU), Fuzhou, Fujian. For pathogen infection, mature single bud stems of YT93-159 and ROC22 with the same growth vigor were syringe-inoculated with 0.5 μL suspension containing 5×10^6^ *Sporisorium scitamineum* spores mL^-1^ according to Su et al (Su et al., 2013). The copy numbers of *S. scitanmineum* in YT93-159 and ROC22 after inoculation were detected using TaqMan real-time fluorescence quantitative PCR according to Su et al (Su et al., 2013) (Table S6). After 48 hours post-inoculation (hpi), five buds from each genotype were excised for immediate protoplast isolation, serving as the experimental group, while the control group was collected immediately after inoculation at 0 h. Sampling for sugarcane tissue-specific and different exogenous hormone treatments was performed as previously described (Zang et al., 2023; Guo et al., 2022). Briefly, four-month-old ROC22 tissue culture seedlings were grown in water for 10 days. Leaves were treated with jasmonate (MeJA), abscisic acid (ABA), and (salicylic acid) SA, with samples collected at specified time intervals (Liu et al., 2018). Additionally, five tissues from mature ROC22 plants, including root, bud, +1 leaf, stem epidermis, and stem pith, were sampled.

**Protoplast preparation**

A reliable protocol for isolating sugarcane bud protoplasts with high viability was established, following the method described by Wang et al (Wang et al., 2021). Initially, the outer skin of the harvested sugarcane buds was carefully peeled off, and the buds were sliced into thin pieces using a sharp blade. Then, they were transferred to a petri dish with 10 mL of freshly prepared enzyme solution (10 mmol L^−1^ MES, pH 5.7, 2% (w/v) Cellulase ‘Onozuka’R-10, 0.5% (w/v) MacerozymeR-10, 10 mmol L^−1^ CaCl_2_, 20 mmol L^−1^ KCl, 0.1% BSA (w/v) and 0.6 mol L^−1^ Mannitol). Subsequently, the tissue slices were immersed in an enzymatic digestion solution and subjected to digestion in a constant temperature shaker set at 25℃ and rotating at 150 rpm for 3 hours in darkness.

After digestion, the protoplasts were filtered through a 40-μm cell sieve, and were collected by centrifuging 150 × *g* for 5 minutes at room temperature to collect them. Following this, the protoplasts underwent two to three washes with WI solution (0.5 M mannitol, 20 mM KCl, 4 mM MES). They were then centrifuged at 150 × *g* for 5 minutes and resuspended in 50 μl ddH_2_O. The activity of single-cell suspensions was measured using fluorescein diacetate and trypan blue staining. The cell concentration was determined using a hemocytometer and a light microscope. Protoplasts with activity levels exceeding 80% were selected for further investigation.

**scRNA-seq library construction, sequencing and data processing**

Single-cell suspensions were processed using the 10× Chromium Controller according to the manufacturer’s instructions for the 10× Genomics Chromium Single-Cell 3’ kit (V3) to generate single-cell gel beads in emulsion. Two independent replicates were conducted. Subsequently, cDNA amplification and library construction were carried out following the standard protocol. The libraries were sequenced using an Illumina NovaSeq 6000 sequencing system (paired-end multiplexing run, 150 bp) by Guangzhou Genedenovo Biotechnology Co., Ltd. The reference genome was aligned and analyzed using the sugarcane R570 genome (Healey et al., 2024).

The raw scRNA-seq datasets were analyzed with Cell Ranger 3.1.0 (10× Genomics). Prior to analysis, cell with high numbers of unique molecular identifiers (UMIs ≥ 8000) or a mitochondrial gene percent equal to or exceeding 10% were filtered out. The raw count matrices were imported into Seurat version 3.1.1 for downstream analysis (Butler et al., 2018). Briefly, we first normalized the data using the NormalizeData function (LogNormalize method, scaling factor of 10,000). Next, we identified variable genes with the FindVariableGenes function (vst method, 2000 features), scaled the data using the ScaleData function, and performed PCA with the RunPCA function (50 principal components). The statistical significance of the PCA scores was assessed using the JackStraw function. We then constructed the SNN graph, clustered the cells using the Louvain algorithm (FindNeighbors and FindClusters), and visualized the data with the non-linear dimensional reduction algorithm RunUMAP (Becht et al., 2018). Marker genes were selected based on the following parameters: the genes expressed in more than 25% of the cells in a cluster, average log_2_ fold change of up-regulated genes greater than 0.58 and *P* value smaller than 0.05.

**Differentially expressed genes and functional enrichment**

Differentially expressed genes (DEGs) were identified using the Find Markers function in Seurat. A significance threshold of *p* < 0.05 and a fold change threshold of |log_2_ fold change| > 0.58 were applied to determine significant differential expression. Gene ontology (GO) and Kyoto Encyclopedia of Genes and Genomes (KEGG) pathway analyses were conducted using the cluster Profiler package (version: 3.18.0) (Yu et al., 2012).

**Pseudo**-**time trajectory**

Monocle2 was employed for trajectory inference and pseudo-time analysis to discern cell clusters (Trapnell et al., 2014). The differentialGene Test function of the Monocle2 package was used to select ordering genes that were likely to be informative in the ordering of cells along the pseudo-time trajectory. Dimensionality reduction clustering was carried out with the reduce dimension function. The orderCells function with default parameters was applied to determine the state transitions of individual cells. Single-cell trajectory analysis was performed using a matrix of cells and gene expressions with Monocle (Version 2.10.1). Specifically, Monocle reduced the dimensionality of the data to a two-dimensional space and ordered the cells (sigma = 0.001, lambda = NULL, param.gamma = 10, tol = 0.001). This allowed us to visualize the trajectory in the reduced dimensional space. Clustering and visualization of gene expression along branch points were executed using the plot_genes_branched_heatmap function.

**Sequence characterization and expression of *ScNPR3* gene**

Based on the identified SoffiXsponR570.01Dg034600.v2.1_NPR3 gene sequence, the cDNA of sugarcane cultivar ROC22 was used as a template to amplify the *NPR3* gene, designated as *ScNPR3* (GenBank accession number: OQ703028), using specific primers (Table S6). A cDNA yeast library of sugarcane inoculated with smut pathogen was constructed by Shanghai Oebiotech Technology Co., Ltd. (Oebiotech, China). Screening with ScNPR3 protein as bait led to the identification of a TGA homologous protein, named ScTGA2, which could interact with ScNPR3. Total RNA extraction and cDNA synthesis were performed following our previous study (Zang et al., 2022). The open reading frame (ORF) of the *ScNPR3* gene was predicted using the ORF Finder (<https://www.ncbi.nlm.nih.gov/orffinder/>), and the conserved domain was identified using the Conserved Domains database (<http://www.ncbi.nlm.nih.gov/Structure/cdd/wrpsb.cgi>). Sequence homology analysis was conducted using DNAMAN 6.0 software. The tissue expression and the relative expression of the *ScNPR3* gene under ABA, SA, and MeJA stresses was carried out by real-time quantitative PCR (RT-qPCR) with specific primers ScNPR3-q (Table S6).

**Vector construction**

The full-length coding sequences (CDS) of ScNPR3 and ScTGA2 were cloned into the entry vector pDONR207 and then transferred into different gateway binary vectors via LR reactions (Thermo Scientific, USA). For subcellular localization and overexpression assays, the coding regions of the *ScNPR3* and *ScTGA2* were cloned into the pFAST-R05-GFP and pEarleyGate-203 vectors, respectively. Bimolecular fluorescence complementation (BiFC), luciferase (LUC), and yeast two-hybrid (Y2H) assays were conducted by cloning the coding regions of *ScNPR3* and *ScTGA2* into the pEarleyGate201-YN, pEarleyGate202-YC, pPGCL, pPGNL, pGADT7 and pGBKT7 vectors, respectively. All primers used for plasmid constructions were listed in Table S6.

**Transient overexpression of *ScNPR3* in *Nicotiana benthamiana***

The *Agrobacterium*-mediated overexpression method was used to transiently overexpress pEarleyGate-203 (control) and the recombinant vector pEarleyGate-203-ScNPR3 in *N. benthamiana* leaves (Sun et al., 2023, 2024). The fungal pathogen *Fusarium solani* var. *coeruleum* was inoculated onto the *N. benthamiana* leaves one day after transient overexpression, and phenotypic changes in the leaves were observed over time. Phenotypic observations and photographs were recorded at 1 day post-inoculation (dpi) and 8 dpi. Adobe Photoshop 2020 was utilized to measure the area of leaf lesions, and photos were captured to record the fluorescence of the leaves under ultraviolet light. Additionally, 3, 3-diaminoben-zidine (DAB) staining was employed to detect H_2_O_2_ in *N. benthamiana* leaves according to our previous studies (Wu et al., 2023). The expression of immune-related marker genes was evaluated by RT-qPCR referred to our previous studies (Sun et al., 2023, 2024; Wang et al., 2024; Zhang et al., 2024). *NtPR-1a/c* and *NtPR2* of SA pathway and *NtHSR203* and *NtHSR515* of hypersensitive response (HR) pathway were selected in this study. All primers of these marker genes were listed in Table S6.

**Genetic transformation of *ScNPR3* in *Nicotiana benthamiana***

The GV3101 strain carrying pEarleyGate203-ScNPR3 was transformed into *N. benthamiana* by the leaf-disk method (Müller et al., 1987). Initial transgenic lines of *N. benthamiana* were selected based on glyphosate resistance, resulting in the identification of 10 transgenic lines, which were subsequently confirmed by PCR with specific primers (Table S6). In addition, seeds from the T_1_ and T_2_ lines were acquired, and homozygous T_2_ lines were used for functional assays of genes under investigation. Disease resistance of transgenic plants was assayed as previously described (Wu et al., 2023).

**RNA-seq and data analysis**

RNA-seq was employed to analyze the alterations in the transcriptome of WT and *ScNPR3*-OE3 plants post *F. solani* var. *coeruleum* inoculation. And cDNA libraries were constructed from WT and *ScNPR3*-OE3 plants that were inoculated with *F. solani* var. *coeruleum* for 0 dpi and 2 dpi, respectively. A total of 12 cDNA libraries were constructed, consisting of WT-CK, WT-T, ScNPR3-CK, and ScNPR3-T with three biological replicates. The sequencing was performed using the NovaSeq 6000 platform (Illumina Inc., USA) by Biomarker Technologies (Beijing, China). The analysis procedure for transcriptomic data was performed as described previously (Sun et al., 2023, 2024; Zhang et al., 2024; Zou et al., 2024).

**Subcellular localization and yeast autoactivation activity test**

To investigate subcellular localization, *Agrobacterium* GV3101 cells containing the 35S:ScNPR3-GFP or 35S:ScTGA2-GFP constructs were cultured overnight in LB liquid medium supplemented with 50 μg/mL kanamycin and 25 μg/mL rifampicin. The bacterial cells were then harvested by centrifugation, and the resulting supernatant was re-suspended in an induction medium consisting of 10 mM MgCl_2_, 10 mM MES (pH 5.7), and 200 mM acetosyringone. Subsequently, the appropriate amount of GV3101 cells was infiltrated into the leaves of *N. benthamiana* plants. Images were taken at 48 h by a laser scanning confocal microscope (TCS SP8, Leica Microsystems, Wetzlar, Germany). The yeast autoactivation activity assay was conducted (Ren et al., 2022), using the Yeast Maker Yeast Transformation System 2 (Takara, USA) and following the manufacturer’s instructions.

**Bimolecular fluorescence complementation**

For BiFC assays, GV3101 cells containing YN-ScNPR3 constructs were mixed with YC-ScTGA2 constructs at a 1:1 ratio and infiltrated into *N. benthamiana* leaves (Liu et al., 2024). Negative controls were also included, where separate leaves were co-transfected with the vector combinations YN-ScNPR3+YC, YN+ YC-ScTGA2, and YN+YN. Images were captured at 48 h by a laser scanning confocal microscope (TCS SP8, Leica Microsystems, Wetzlar, Germany) with an excitation wavelength and emission filter of 510 nm.

**Luciferase complementation assay**

The LUC assay was conducted using a tobacco transient expression system. GV3101 cells carrying 35S: ScNPR3-LUC^C^ and 35S: ScTGA2-LUC^N^ plasmids were mixed in equal proportions and co-infiltrated into *N. benthamiana* leaves. The leaves were then incubated in a growth room for 48 h (Yang et al., 2023). After that, the leaves were sprayed with 1 mM luciferin and incubated in darkness at room temperature for 10 minutes. Luminescence was recorded using a low-light cooled charge-coupled device (CCD) camera (PerkinElmer, USA), and the intensity of luminescence was quantified using the Image J software.

**Yeast two-hybrid assay**

To validate protein interactions, the coding regions of *ScNPR3* and *ScTGA2* were cloned into the pGADT7 and pGBKT7 vectors, respectively. This resulted in the creation of bait and prey vectors, namely AD-ScNPR3, AD-ScTGA2, BD-ScNPR3, and BD-ScTGA2. These bait and prey vectors were co-transformed into Y2HGold chemically competent cells. The well-grown transformants were diluted with sterilized ddH_2_O and selected on synthetic dropout (SD) agar medium without Trp and Leu (SD/-Leu/-Trp). Subsequently, they were grown on SD/-Trp-Leu-His-Ade agar medium (Ling et al., 2022).

**Supplemental References**

Becht, E., McInnes, L., Healy, J., Dutertre, C. A., Kwok, I. W. H., Ng, L. G., Ginhoux, F., and Newell, E. W. (2018). Dimensionality reduction for visualizing single-cell data using UMAP. *Nat. Biotechnol*. 37:38-44.

Butler, A., Hoffman, P., Smibert, P., Papalexi, E., and Satija, R. (2018). Integrating single-cell transcriptomic data across different conditions, technologies, and species. *Nat. Biotechnol*. 36:411-420.

Guo, X., Liang, J., Lin, R., Zhang, L., Zhang, Z., Wu, J., and Wang, X. (2022). Single-cell transcriptome reveals differentiation between adaxial and abaxial mesophyll cells in *Brassica* *rapa*. *Plant Biotechnol. J*. 20:2233-2235.

Healey, A. L., Garsmeur, O., Lovell, J. T., Shengquiang, S., Sreedasyam, A., Jenkins, J., Plott, C. B., Piperidis, N., Pompidor, N., Llaca, V.*,* et al. (2024). The complex polyploid genome architecture of sugarcane. *Nature* 628:804-810.

Ling, H., Fu, X., Huang, N., Zhong, Z., Su, W., Lin, W., Cui, H., and Que, Y. (2022). A sugarcane smut fungus effector simulates the host endogenous elicitor peptide to suppress plant immunity. *New Phytol*. 233:919-933.

Liu, F., Huang, N., Wang, L., Ling, H., Sun, T., Ahmad, W., Muhammad, K., Guo, J., Xu, L., Gao, S., et al. (2017). A Novel L-ascorbate peroxidase 6 gene, *ScAPX6*, plays an important role in the regulation of response to biotic and abiotic stresses in sugarcane. *Front. Plant Sci* 8:2262.

Liu, K., Shi, L., Luo, H., Zhang, K., Liu, J., Qiu, S., Li, X., He, S., and Liu, Z. (2024). *Ralstonia solanacearum* effector RipAK suppresses homodimerization of the host transcription factor ERF098 to enhance susceptibility and the sensitivity of pepper plants to dehydration. *Plant J*. 117:121-144.

Müller, A. J., Mendel, R. R., Schiemann, J., Simoens, C., and Inzé, D. (1987). High meiotic stability of a foreign gene introduced into tobacco by *Agrobacterium*-mediated transformation. *Mol. Gen. Genet*. 207:171-175.

Ren, Y., Zou, W., Feng, J., Zhang, C., Su, W., Zhao, Z., Wang, D., Sun, T., Wang, W., and Cen, G. (2022). Characterization of the sugarcane MYC gene family and the negative regulatory role of *ShMYC4* in response to pathogen stress. *Ind. Crop Prod*. 176:114292.

Su, Y., Wang, S., Guo, J., Xue, B., Xu, L., and Que, Y. (2013). A TaqMan real-time PCR assay for detection and quantification of *Sporisorium scitamineum* in sugarcane. *Sci. World J*. 2013:942682.

Sun, T., Chen, Y., Feng, A., Zou, W., Wang, D., Lin, P., Chen, Y., You, C., Que, Y., and Su, Y. (2023). The allene oxide synthase gene family in sugarcane and its involvement in disease resistance. *Ind. Crop Prod*. 192:116-136.

Sun, T., Wu, Q., Zang, S., Zou, W., Wang, D., Wang, W., Shen, L., Zhang, S., Su, Y., and Que, Y. (2024). Molecular insights into OPR gene family in *Saccharum* identified a *ScOPR2* gene could enhance plant disease resistance. *Plant J*. 120(1):335-353.

Trapnell, C., Cacchiarelli, D., Grimsby, J., Pokharel, P., Li, S., Morse, M., Lennon, N. J., Livak, K. J., Mikkelsen, T. S., and Rinn, J. L. (2014). The dynamics and regulators of cell fate decisions are revealed by pseudotemporal ordering of single cells. *Nat. Biotechnol*. 32:381-386.

Wang, Q., Yu, G., Chen, Z., Han, J., Hu, Y., and Wang, K. (2021). Optimization of protoplast isolation, transformation and its application in sugarcane (*Saccharum spontaneum* L). *Crop J*. 9:133-142.

Wang, D., Wang, W., Zang, S., Qin, L., Liang, Y., Lin, P., Su, Y., and Que, Y. (2024). Sugarcane transcription factor ScWRKY4 negatively regulates resistance to pathogen infection through the JA signaling pathway. *Crop J*. 12:164-176.

Wu, Q., Chen, Y., Zou, W., Pan, Y. B., Lin, P., Xu, L., Grisham, M. P., Ding, Q., Su, Y., and Que, Y. (2023). Genome-wide characterization of sugarcane catalase gene family identifies a *ScCAT1* gene associated disease resistance. *Int. J. Biol. Macromol*. 232:123398.

Yang, S., Cai, W., Wu, R., Huang, Y., Lu, Q., Hui, W., Huang, X., Zhang, Y., Wu, Q., Cheng, X.*,* et al. (2023). Differential CaKAN3-CaHSF8 associations underlie distinct immune and heat responses under high temperature and high humidity conditions. *Nat. Commun*. 14:4477.

Yu, G., Wang, L. G., Han, Y., and He, Q. Y. (2012). clusterProfiler: an R package for comparing biological themes among gene clusters. *OMICS*. 16:284-287.

Zang, S., Qin, L., Zhao, Z., Zhang, J., Zou, W., Wang, D., Feng, A., Yang, S., Que, Y., and Su, Y. (2022). Characterization and functional implications of the nonexpressor of pathogenesis-related genes 1 (*NPR1*) in *Saccharum*. *Int. J. Mol. Sci*. 23:7984.

Zang, Y., Pei, Y., Cong, X., Ran, F., Liu, L., Wang, C., Wang, D., and Min, Y. (2023). Single-cell RNA-sequencing profiles reveal the developmental landscape of the *Manihot esculenta Crantz* leaves. *Plant Physiol*. 194:456-474.

Zhang, C., Li, Z., Sun, T., Zang, S., Wang, D., Su, Y., Wu, Q., and Que, Y. (2024). Sugarcane *ScCAX4* is a negative regulator of resistance to pathogen infection. *J. Agric. Food Chem*. 72:13205-13216.

Zou, W., Sun, T., Chen, Y., Wang, D., You, C., Zang, S., Lin, P., Wu, Q., Su, Y., and Que, Y. (2024). Sugarcane *ScOPR1* gene enhances plant disease resistance through the modulation of hormonal signaling pathways. *Plant Cell Rep*. 43:158.

**Supplemental Figures**


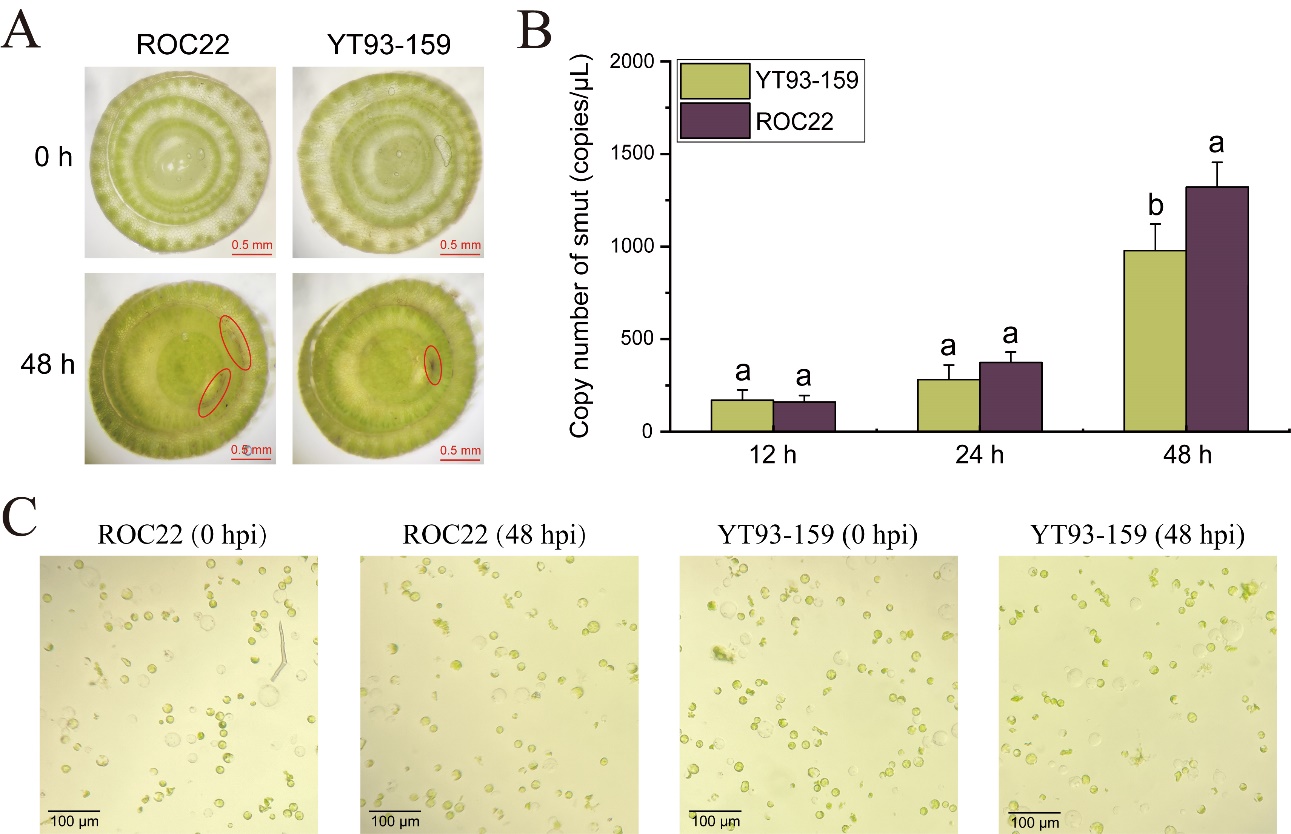


**Figure S1.** Quantitative assessment of *S. scitamineum* infection and protoplast isolation from buds of ROC22 and YT93-159 at 0 and 48 hpi. (A) Microscopic observation of cross-sectional views of ROC22 and YT93-159 sugarcane buds at 0 and 48 hpi. Red elliptical circles indicated pathological changes in sugarcane bud tissue after inoculation. Bar = 0.5 mm. (B) Quantification of the pathogen in YT93-159 and ROC22 buds infected with *S. scitamineum*. Five single buds were mixed as one biological sample, and three biological replicates were performed. (C) Protoplast isolation from YT93-159 and ROC22 buds inoculated with smut at 0 and 48 hpi. The protoplasts shown were isolated approximately 3 hours after tissue sampling at 0 and 48 hpi. hpi, hour post-infection.


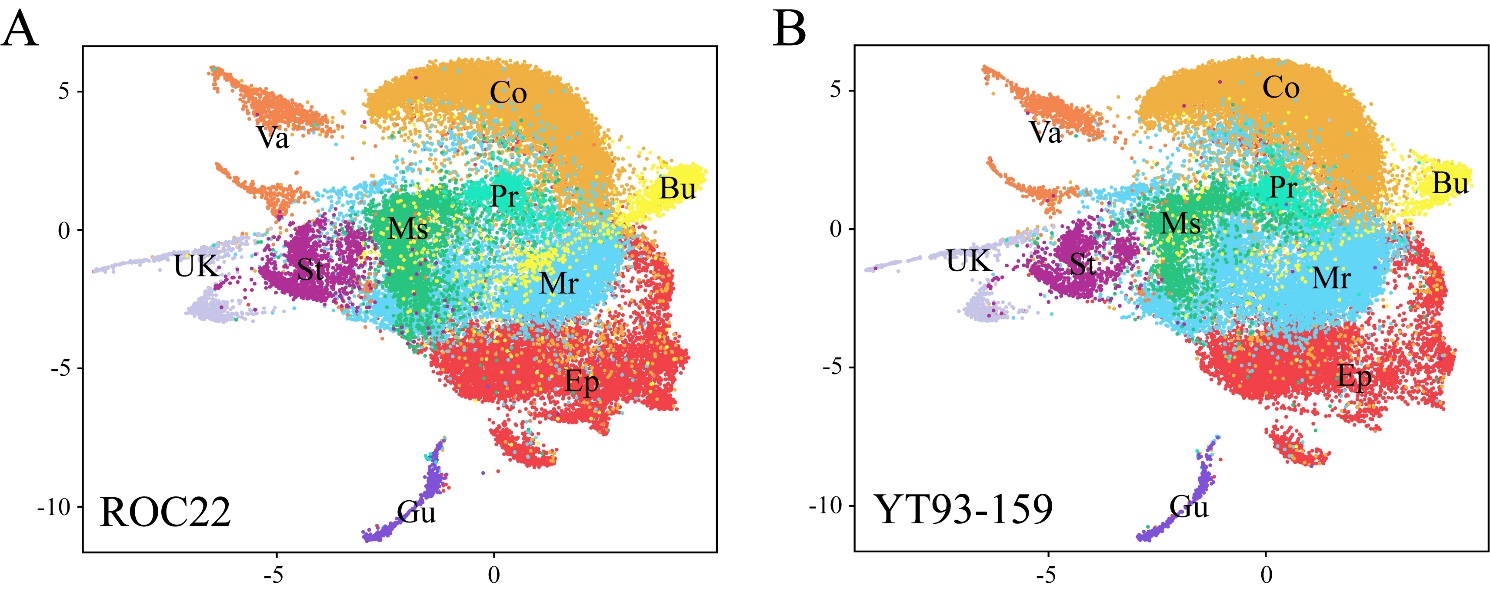


**Figure S2.** UMAP visualization of the 10 major cell clusters in ROC22 (A) and YT93-159 (B).


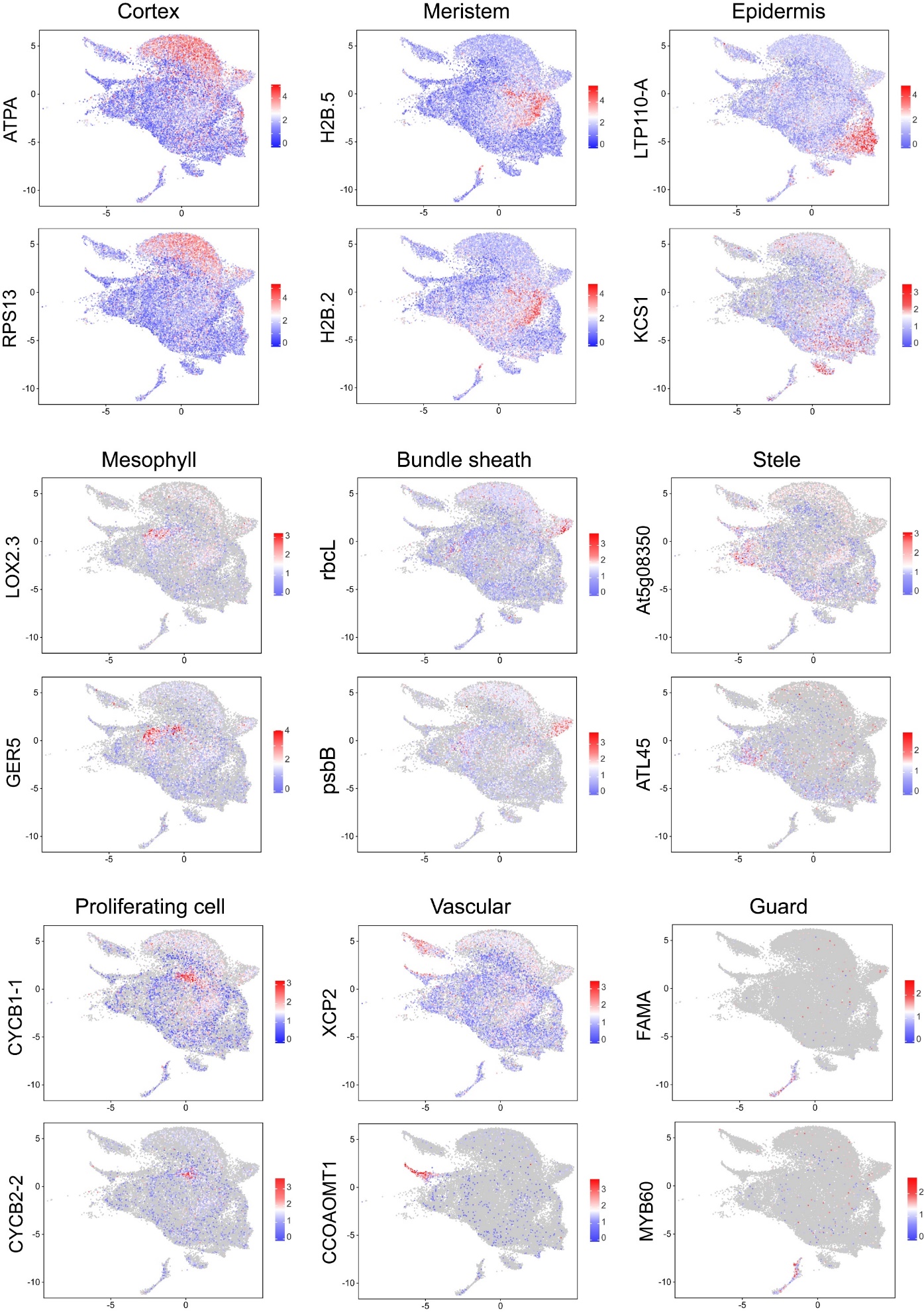


**Figure S3.** UMAP plot showing the distribution of marker genes used to identify cell types in sugarcane buds.


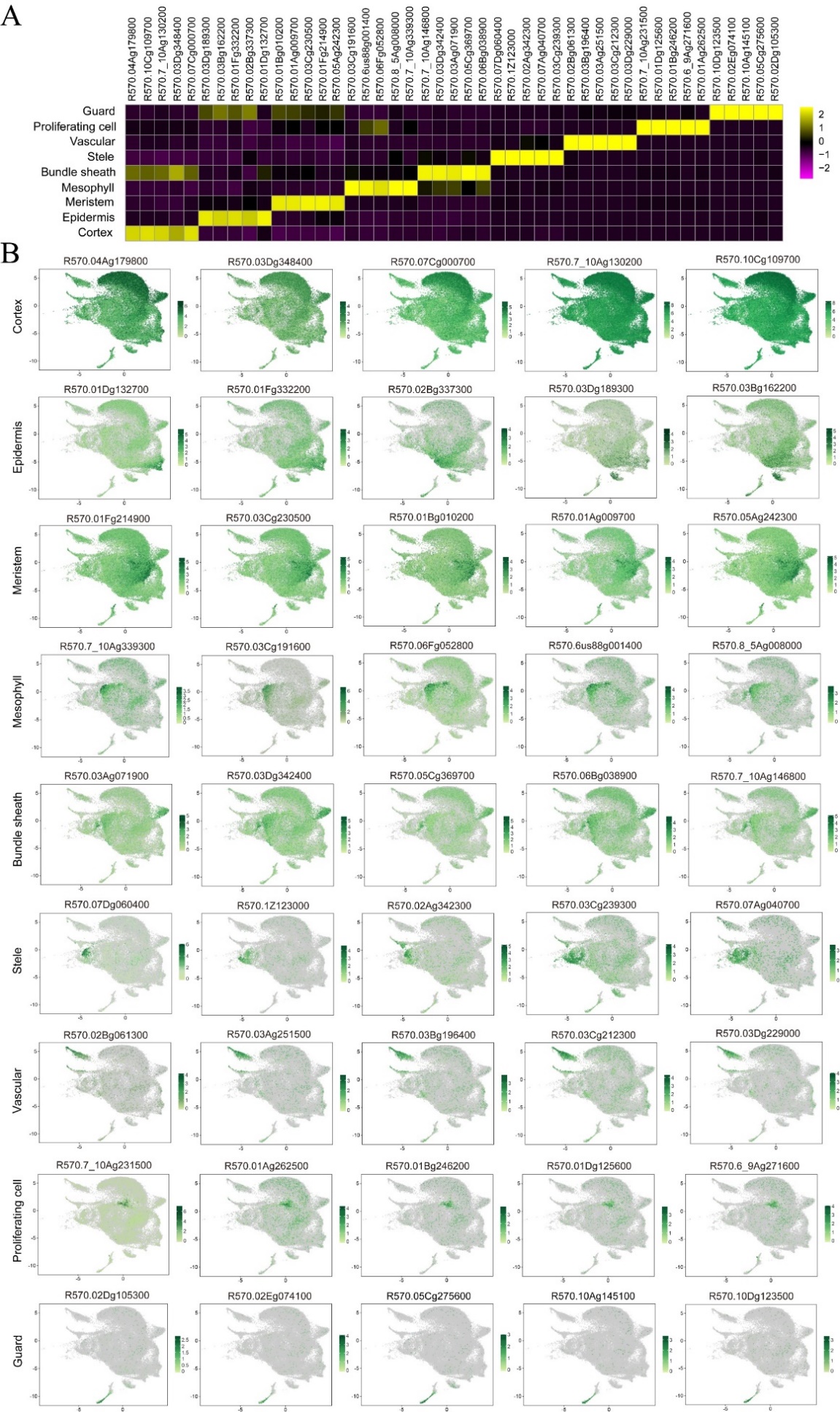


**Figure S4.** Identification of new sugarcane marker genes in the clusters corresponding to the nine recognized cell types. (A) The top five DEGs with the highest expression levels (log_2_ fold change) in each sub-cluster. (B) The expression patterns of nine new marker genes distributed in the UMAP plots.


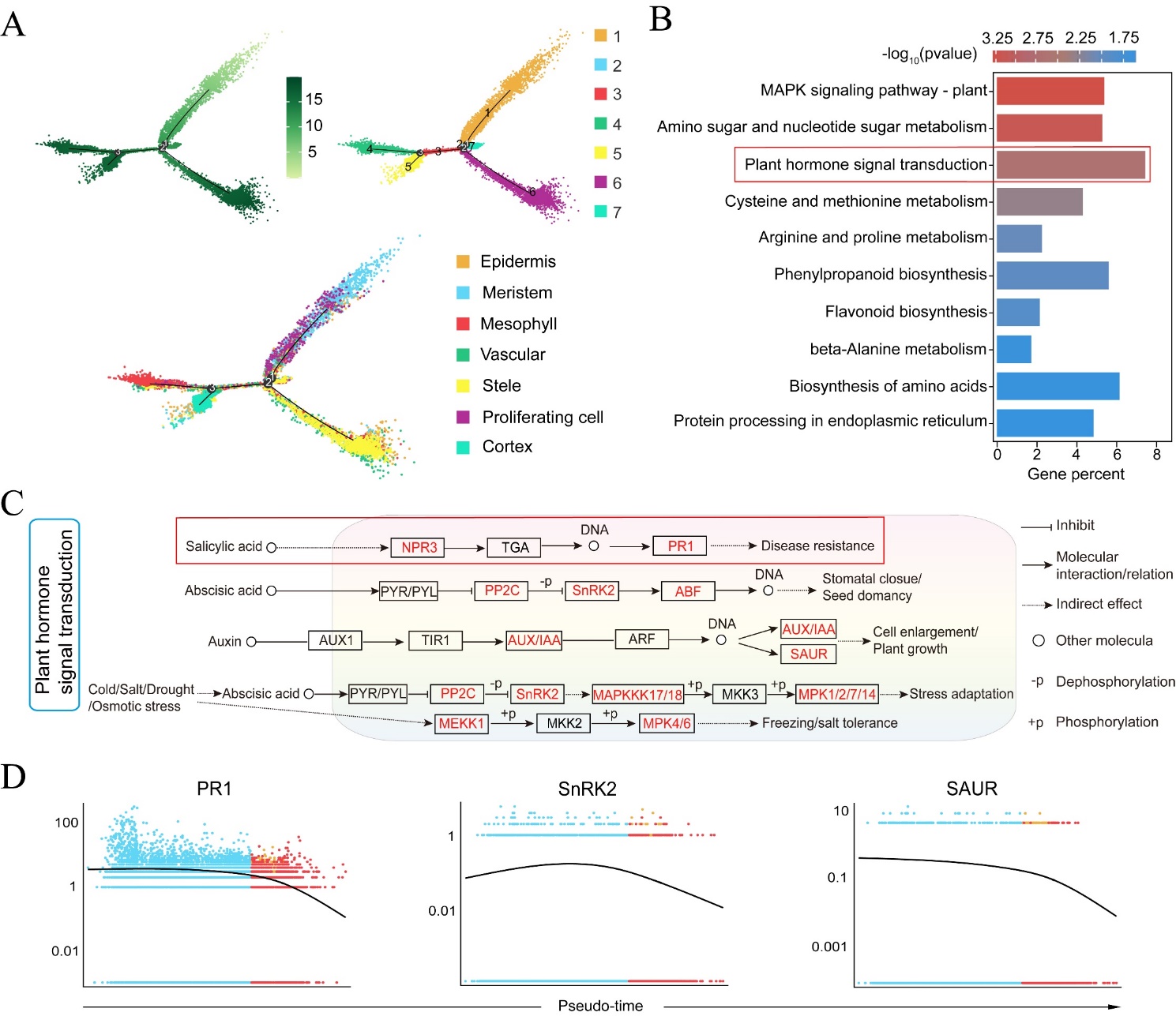


**Figure S5.** Pseudo-time trajectory of meristem cell differentiation into cortex cells and proliferating cells in sugarcane. (A) Pseudo-temporal trajectories of seven cell types in sugarcane buds. The cell ordering along the differentiation trajectory was presented by pseudo-time states, samples, and cell types. (B) The top 10 KEGG enrichment pathways of significantly altered genes. The red boxes indicated candidate KEGG pathway. (C) The plant hormone signal transduction signaling pathways. The red font indicated the candidate DEGs. The red box indicated the candidate regulatory pathway. (D) Representative genes related to plant hormone signal transduction were selected to illustrate their expression patterns before and after cell differentiation.


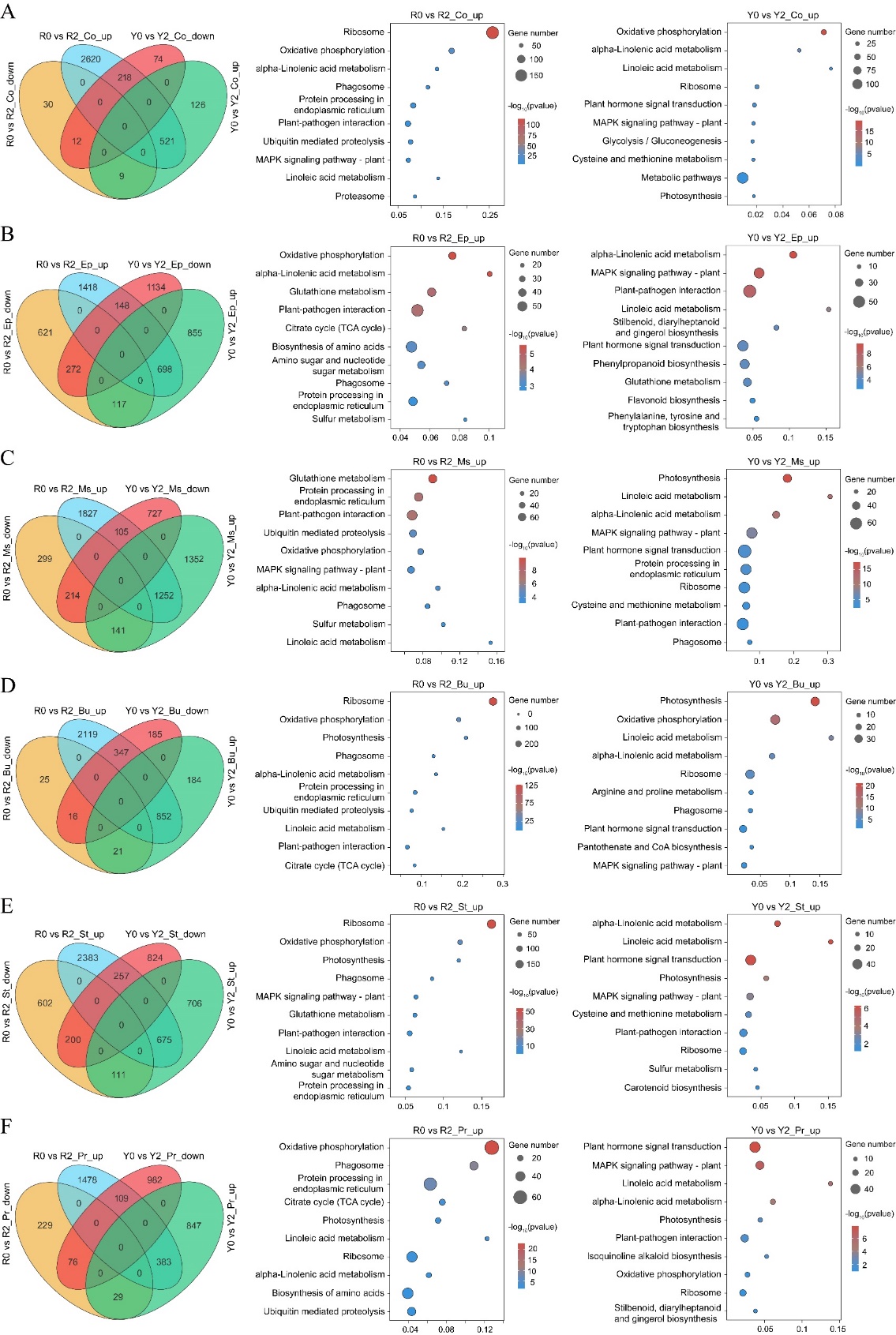


**Figure S6.** Comparative analysis of six cell types in ROC22 and YT93-159 at 0 d and 2 d post inoculation. (A-F) Venn diagrams of all genes exhibiting up- or down-regulated expression between R0_vs_R2 and Y0_vs_Y2 in the clusters of cortex (A), epidermis (B), mesophyll (C), bundle sheath (D), stele (E), and proliferative cells (F). (A-F) KEGG enrichment analysis of up-regulated genes in the cortex (A), epidermis (B), mesophyll (C), bundle sheath (D), stele (E), and proliferative cell (F) clusters between R0_vs_R2 and Y0_vs_Y2. Co: cortex; Ep: epidermis; Ms: mesophyll; Bu: bundle sheath; St: stele; Pr: proliferative cell. R0, R2, Y0, and Y2 represented samples of ROC22-0d, ROC22-0d, YT93-159-0d, and YT93-159-2d, respectively. DEG sets were named R0_vs_R2 and Y0_vs_Y2.


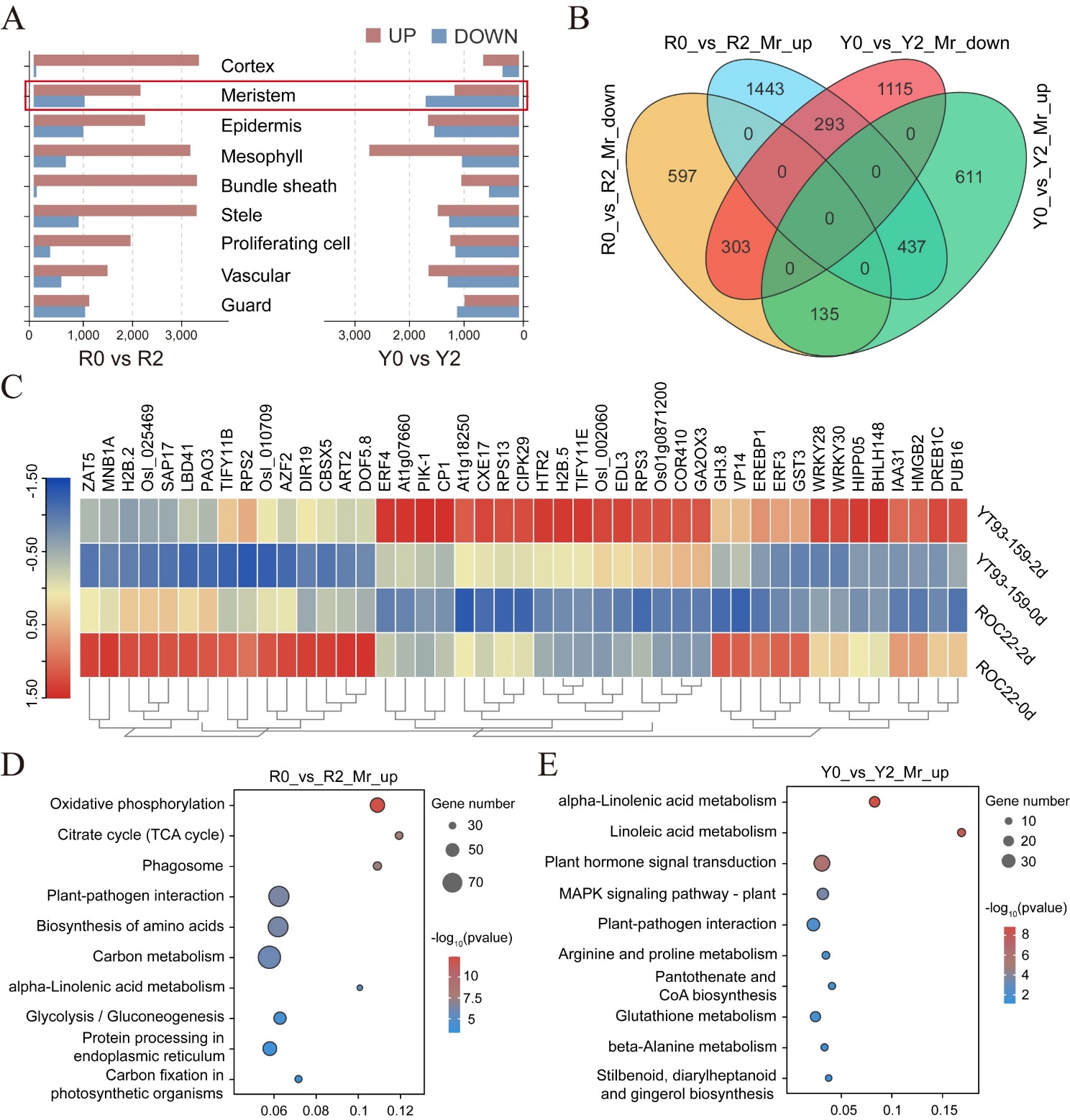


**Figure S7.** Highly conserved cell-type clusters and heterogeneity genes between ROC22 and YT93-159. (A) The number of DEGs in each cell type in ROC22 and YT93-159. (B) Venn diagrams of all genes exhibiting up- or down-regulated expression between R0_vs_R2 and Y0_vs_Y2 in meristem cluster. (C) Expression profiles of genes down-regulated in ROC22 and up-regulated in YT93-159 in meristem after inoculation. (D) KEGG enrichment of up-regulated genes in the meristem cluster in R0_vs_R2. (E) KEGG enrichment of up-regulated genes in the meristem cluster in Y0_vs_Y2. R0, R2, Y0, and Y2 represented samples of ROC22-0d, ROC22-0d, YT93-159-0d, and YT93-159-2d, respectively.


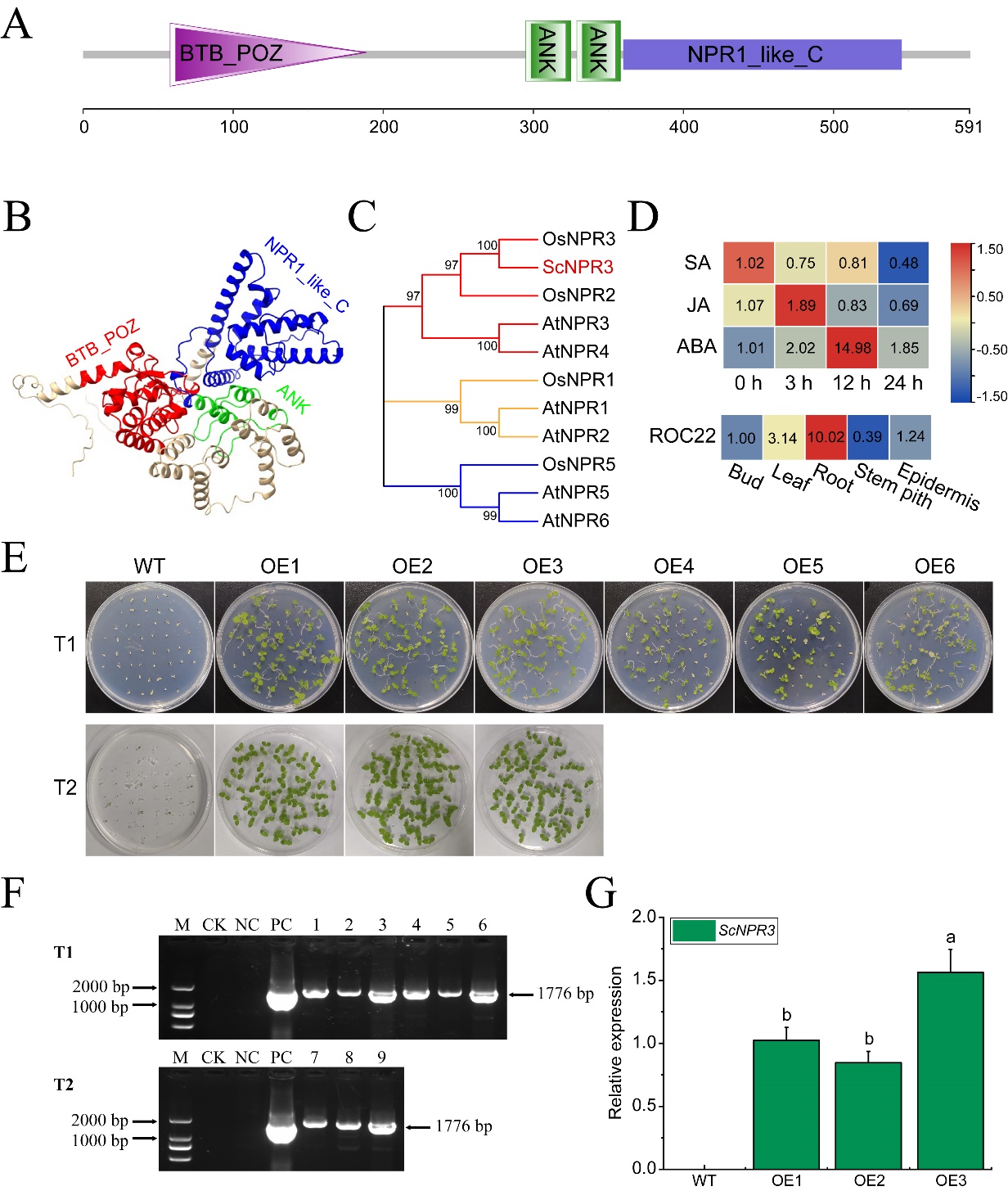


**Figure S8.** Characterization of the *ScNPR3* gene and generation of transgenic *Nicotiana benthamiana* plants. (A) Conserved domains of ScNPR3 protein. (B) 3D structure diagram of ScNPR3 protein based on Alphafold2 homology modeling. (C) A phylogenetic tree of the NPR protein. ScNPR3 protein was shown in red font. (D) Expression patterns of *ScNPR3* gene in different sugarcane tissues and under SA, MeJA, and ABA stresses. (E) Screening of the T_1_ and T_2_ generation plants of transgenic *N. benthamiana* overexpressing *ScNPR3*. (F) PCR detection result of genomic DNA in T_1_ and T_2_ generation of transgenic *Nicotiana benthamiana* plants overexpressing *ScNPR3.* CK: blank control; NC: negative control, PCR products of the WT *N. benthamiana*; PC: positive control, PCR products of the overexpression vector pEarleyGate-203-*ScNPR3*; 1-9: PCR products of the transgenic *N. benthamiana*. (G) Relative expression levels of *ScNPR3* in transgenic plants by RT-qPCR. Different lowercase letters on the bars indicated a significant difference, as determined by Duncan’s new multiple range test (*P* < 0.05). All data points were means ± standard deviation (*n =* 3). WT represented wild-type *N. benthamiana* and OE represented *ScNPR3* overexpressing transgenic lines.


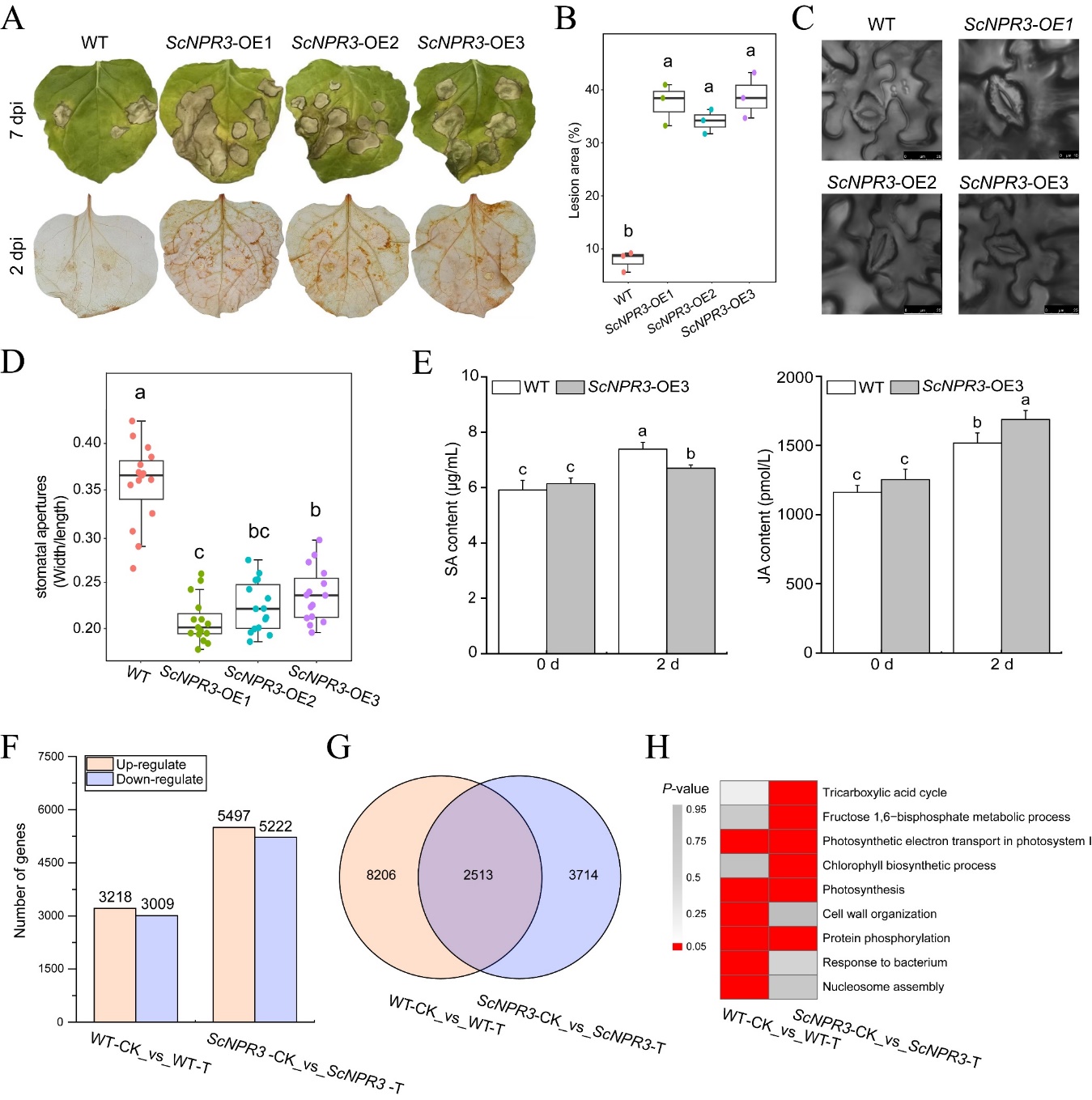


**Figure S9.** Disease resistance and transcriptome assessment of *ScNPR3* transgenic *N. benthamiana* inoculated with *F. solani* var. *coeruleum*. (A) The disease symptoms at 7 dpi and DAB staining at 2 dpi of *N*. *benthamiana* leaves inoculated with *F*. *solani* var. *coeruleum*. dpi: days post inoculation. (B) Lesion area of leaves after infection with *F. solani* var. *coeruleum* for 7 d. (C, D) Phenotype of the guard cells (C) and stomatal aperture (D) in the leaves of *N. benthamiana* plants inoculated with *F*. *solani* var. *coeruleum* at 2 dpi. Bars = 25 μm. Dots with different colors represented three independent experiments with five biological replicates in each experiment (n = 15). (E) Determination of SA and JA contents in WT and OE plants at 0 dpi and 2 dpi. All data points were means ± standard error (n = 3). Different letters above the columns indicated significant differences (Duncan’s new multiple range test at *P* < 0.05). (F) DEGs between *ScNPR3* overexpressing transgenic lines and WT *N. benthamiana* plants inoculated with *Fusarium solani* var. *coeruleum* for 0 d (CK) and 2 d (T). WT-CK_vs_WT-T and ScNPR3-CK_vs_ScNPR3-T represented the number of DEGs at 0 dpi and 2 dpi, respectively. (G) Venn diagrams of DEGs between WT-CK_vs_WT-T and *ScNPR3*-CK_vs_ *ScNPR3*-T. (H) GO enrichment analysis of seven important biological processes of the unique DEGs of WT-CK_vs_WT-T and ScNPR3-CK_vs_ ScNPR3-T. *P*-value < 0.05 was used as the threshold for significantly enriched GO terms.


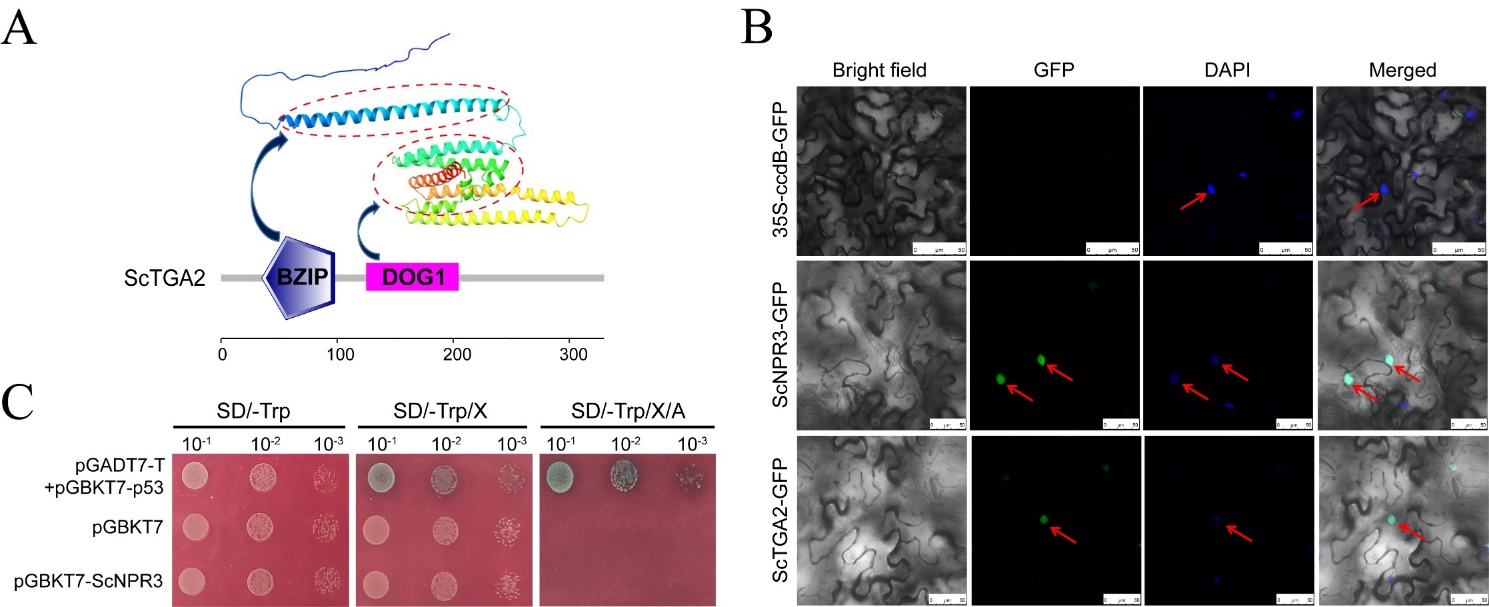


**Figure S10.** The subcellular localization of ScNPR3 and ScTGA2 and the transcriptional self-activation activity of ScNPR3. (A) Structure and conserved domains of ScTGA2. Aligning the conserved domains using ChimeraX-1.5. (B) Subcellular location of ScNPR3 and ScTGA2 proteins in *N. benthamiana*. Images were captured using visible light, green fluorescence, blue fluorescence, and merged light. pFAST-R05 vector expressed a gene fused to a green fluorescent protein (*GFP*) gene, which followed the *ccdB* gene (with a stop codon). DAPI: 4′, 6-diamidino-2-phenylindole. Red arrows indicated nucleus. Bar = 50 µm. (C) Transcriptional self-activating activity of ScNPR3 proteins in yeast cells. SD/-Trp, synthetic dropout medium without tryptophan; SD/-Trp/X, SD/-Trp with 5-bromo-4-chloro-3-indoxyl-α-D-Galactopyranoside; SD/-Trp/X/A, SD/-Trp/X with aureobasidin A; pGADT7-T+pGBKT7-p53, positive control; pGBKT7, empty vector control; pGBKT7-ScNPR3, yeast cells transformed with *ScNPR3* genes. 10^-1^-10^-3^ represented a dilution of the yeast solution by a factor of 10 to 1000.
